# Synchronization hubs may arise from strong rhythmic inhibition during gamma oscillations in primary visual cortex

**DOI:** 10.1186/1471-2202-12-S1-P277

**Published:** 2011-07-18

**Authors:** Stefanos E Folias, Danko Nikolić, Jonathan E Rubin

**Affiliations:** 1 Department of Mathematics, University of Pittsburgh, Pittsburgh, PA, USA; 2 Department of Neurophysiology, Max Planck Institute for Brain Research, Wolfgang Goethe University, 60528 Frankfurt/Main, Germany; 3 Frankfurt Institute for Advanced Studies, Wolfgang Goethe University, 60438 Frankfurt/Main, Germany

## 

Parallel multiunit recordings from V1 in anesthetized cat were collected during the presentation of randomÂ sequences of drifting sinusoidal gratings at 12 fixed orientations while gamma oscillations were present. In agreement with the seminal work [1], most units were orientation selective to varying degrees and synchronization was evident in spike trainÂ crosscorrelograms computed between units with similar preferred orientations, particularly during theÂ presentation of optimal stimuli. Interestingly, a subset of units, which we refer to as synchronization hubs, wereÂ additionally found to synchronize with units having differing preferred orientations which was consistentÂ with a previous study [2]. Moreover, oscillatory patterning in spike train autocorrelograms was alsoÂ found to be strongest in units denoted as synchronization hubs, and synchronization hubs also tended to have narrower tuning curves relative to other units.

We used simplified computational models of small networks of V1 neurons to demonstrate that neurons subject to a sufficiently strong level of inhibitory input can function as synchronization hubs. Neurons were endowedÂ either with integrate-and-fire or conductance-based dynamics and each neuron received a combinationÂ of excitatory (AMPA) synaptic inputs that were Poisson-distributed and inhibitory (GABA) inputs thatÂ were coherent at a gamma-frequency range. If the strength of rhythmic inhibition was increased for aÂ subset of neurons in the network, and excitation was increased simultaneously to maintain a fixed firingÂ rate, then these neurons produced stronger oscillatory patterning in their discharge probabilities. TheÂ oscillations in turn synchronized these neurons with other neurons in the network. Importantly, theÂ strength of synchronization increased with neurons of differing orientation preferences even though noÂ direct synaptic coupling existed between the hubs and the other neurons.

Enhanced levels of inhibition account for the emergence of synchronization hubs in the following way:Â Inhibitory inputs exhibiting a gamma rhythm determine a time window within which a cell is likely toÂ discharge. Increased levels of inhibition narrow down this window further simultaneously leading to (i)Â even stronger oscillatory patterning of the neuron's activity and (ii) enhanced synchronization withÂ other neurons. This enables synchronization even between cells with differing orientation preferences.Â Additionally, the same increased levels of inhibition may be responsible for the narrow tuning curves ofÂ hub neurons. In conclusion, synchronization hubs may be the cells that interact most strongly with theÂ network of inhibitory interneurons during gamma oscillations in primary visual cortex.

## References

[B1] Gray C Singer W Stimulus-specific neuronal oscillations in orientation columns of cat visual cortex Proc. Nati. Acad. Sci 1989 86 1698 1702 10.1073/pnas.86.5.1698 PMC286768 2922407

[B2] Yu S Huang D Singer W Nikolić D A small world of neuronal synchrony. Cerebral Cortex 2008 18 2891 2901 18400792 10.1093/cercor/bhn047 PMC2583154

